# Genome-wide identification of the wall-associated kinase gene family and their expression patterns under various abiotic stresses in soybean (*Glycine max* (L.) Merr)

**DOI:** 10.3389/fpls.2024.1511681

**Published:** 2025-01-16

**Authors:** Xiangnan Li, Sifei Qi, Lingzhi Meng, Peisen Su, Yongwang Sun, Nan Li, Dan Wang, Yinglun Fan, Yong Song

**Affiliations:** ^1^ College of Agriculture and Biology, Liaocheng University, Liaocheng, China; ^2^ Economic Crop Research Institute, Puyang Academy of Agriculture and Forestry Sciences, Puyang, China

**Keywords:** abiotic stresses, expression analysis, genome-wide identification, soybean, wall-associated kinase

## Abstract

The wall-associated kinase (WAK) gene family encodes functional cell wall-related proteins. These genes are widely presented in plants and serve as the receptors of plant cell membranes, which perceive the external environment changes and activate signaling pathways to participate in plant growth, development, defense, and stress response. However, the WAK gene family and the encoded proteins in soybean (Glycine max (L.) Merr) have not been systematically investigated. In this study, the soybean WAK genes (*GmWAK*) were identified based on genome-wide sequence information, the basic characteristics, chromosome location, gene replication, expression pattern, and responses to stress were comprehensively analyzed. A total of 74 *GmWAK* genes were identified and mapped to 19 different chromosomes in the soybean genome. Seventy-four *GmWAK* genes were divided into four groups, and *GmWAK* genes in the same group shared similar gene structures and conserved motifs. Thirty-seven duplicate pairs were identified in 74 *GmWAK* genes. Segmental duplication (SD) was critical in soybean WAK gene family expansion, and purification selection occurred during evolution. The promoter cis-element analysis displayed many hormone- and stress-related response elements in the promoter regions of *GmWAK* genes. *GmWAK* genes were diversely expressed in different organs and tissues, with most actively responding to cold, heat, salt, drought, and heavy metal stresses, suggesting that *GmWAK* genes could exhibit relevant roles in various bioprocesses.

## 1 Introduction

The cell wall is a thick, tough, and slightly elastic structure surrounding the cell membrane. Plant cell walls are composed of cellulose, hemicellulose, pectin, and small amounts of structural proteins, which are critical in maintaining cell morphology and resisting pathogen invasion (Kohorn et al., 2006; Underwood, 2012). Receptor-like kinases (RLKs) are transmembrane proteins located on the cell membrane that act as receptors for signaling molecules which play important regulatory roles in almost all life activities (Soltabayeva et al., 2022; Mehla et al., 2024; Ye et al., 2017). According to the different extracellular domains, RLKs can be divided into more than 40 subfamilies, including WAK-RLKs, Lec-RLKs, LRR-RLKs, etc (Afzal et al., 2008; Lehti-Shiu et al., 2009). Among them, wall-associated kinases (WAKs) comprise a specific RLK gene family associated with the cell wall pectin (Decreux and Messiaen, 2005; Kanneganti and Gupta, 2008). WAK proteins display typical structural domains, including the GUB_WAK_bind domain, epidermal growth factor (EGF) domain, transmembrane domain, and Pkinase domain (He et al., 1999). The GUB_WAK_bind and EGF domains are located outside the cell and assist cells in perceiving external signals (He et al., 1999). The Pkinase domain is located in the cell and is the starting point of the downstream signaling cascade in the cytoplasm (Tocquard et al., 2014; Wagner and Kohorn, 2001; Weremczuk et al., 2020). The *WAK* gene family is a subfamily of RLKs, which act as the information transmission link between the cell wall, cell membrane, and cytoplasm, being important in regulating plant growth, development, and response to environmental stress (Zuo et al., 2015; Sun et al., 2020; Dou et al., 2021).

Currently, the WAK gene families have been identified in a number of species. For instance, 27 WAK genes are present in *Arabidopsis* (*Arabidopsis thaliana*), 125 in rice (*Oryza sativa*), 29 in cotton (*Gossypium hirsutum*), 6 in wheat (*Triticum aestivum*), 68 in rose (*Rosa chinensis*), 44 in apple (*Malus domestica*), 41 in walnut (*Juglans regia*), and 29 in potato (*Solanum tuberosum*) (Verica and He, 2002; Zhang et al., 2005; Dou et al., 2021; Liu et al., 2006, 2021; Zuo et al., 2019; Li et al., 2022; Yu et al., 2022). WAK genes are important in plant growth and development. In *Arabidopsis*, *AtGRP3* interacts with the extracellular domain of the receptor-like kinase *AtWAK1* and inhibits root cell expansion. In addition, its gene knockout mutant shows increasing root length (Kohorn, 2001; Mangeon et al., 2016). The antisense expression of *AtWAK4* inhibits cell elongation and altered root development (Lally et al., 2001). In rice, the wall-associated receptor-like kinase gene *OsDEES1* regulates early embryonic sac development, and RNA interference silencing (RNAi) of *OsDEES1* causes a high rate of female sterility (Wang et al., 2012). *HvWAK1* is specifically expressed in barley roots, and a shorter root was observed in *HvWAK1* Ds mutants than in wild-type specimens (Kaur et al., 2013; Tripathi et al., 2021). *SlWAKL2* is homologous to *AtWAKL14* in *Arabidopsis* and is specifically expressed in tomato vascular tissue. *SlWAKL2* RNAi plants had small fruits, few seeds, and few vascular bundles (Ma et al., 2024).

WAK genes were also involved in the responses to biotic and abiotic stresses. The expression of *OsWAK112* in rice was inhibited by salt stress, and decreased S-adenosyl-L-methionine synthetase (SAMS) content, ethylene content, and plant survival rate were observed in *OsWAK112*-overexpressing plants (Lin et al., 2021). *OsWAK11* was upregulated by heavy metal stress in rice. *OsWAK11* appears to be involved in Cu^2+^ detoxification, mediating Cu^2+^ accumulation in the cell wall, by regulating cell wall methylesterification and alleviating metal ion toxicity (Hu et al., 2014; Xia et al., 2018). *TaWAK20* was a positive regulatory factor of cadmium (Cd) stress, and its overexpression improved the Cd tolerance of transgenic plants by increasing the activities of antioxidant enzymes and H_2_O_2_ content (Li, 2023). *OsWAK2* participates in the signaling pathway of rice blast disease resistance. Transgenic plants overexpressing *OsWAK2* exhibited enhanced disease resistance, whereas RNAi plants showed a loss of resistance (Li et al., 2004). *ZmWAK02* encodes an RD-WAK protein in maize. Transgenic lines, mutants, and complementation lines confirmed that *ZmWAK02* was the resistance gene for maize gray spot disease (Dai et al., 2024).

Soybean (*Glycine max* (L.) Merr) is an important oil crop and a primary source of plant-based proteins for human and animal feed, playing a crucial role in global food security. The soybean yield and quality are affected by abiotic stresses, such as salt, temperature, drought, and heavy metal stress (Nagajyoti et al., 2010; Hussain et al., 2023; Xu et al., 2023). The emergence rate of soybeans decreases by 5%–10% under cold stress (Staniak et al., 2021). The yield, harvest index, and seed quality of soybeans are reduced under heat and drought stress conditions (Carrera and Dardanelli, 2017; Djanaguiraman et al., 2013; Mourtzinis et al., 2015; Rasheed et al., 2022). Salt stress decreases the chlorophyll content and photosynthetic rate of soybeans and inhibits photosynthetic carbon metabolism (Weisany et al., 2011). Under aluminum (Al) stress, the Ca^2+^ homeostasis and signal transduction in soybeans are disrupted, reactive oxygen species (ROS) is increased, membrane peroxidation is accelerated, and root growth of soybeans is restrained (Chandra and Keshavkant, 2021; Chauhan et al., 2021; Ma, 2007). Under Cd stress, the photosynthetic system, cell membrane, and respiratory metabolism are damaged, inhibiting soybean growth and development (Liu et al., 2024; Shi et al., 2019).

The *WAK* gene family was identified in multiple species and was recognized as critical for plant growth, development, and stress tolerance (Verica and He, 2002; Zhang et al., 2005; Dou et al., 2021; Liu et al., 2006, 2021). However, the *WAK* gene family in soybeans has not yet been systematically investigated. In this study, the soybean *WAK* gene family was identified. Domain structure, duplication events, and phylogenetic relationships were analyzed. The tissue-specific expression of these genes was detected using RNA-Seq, and analysis of expression patterns under various abiotic stresses was performed by qRT-PCR. This study will provide potential candidate genes for further functional investigation at the molecular level and the molecular breeding of soybeans with stress tolerance.

## 2 Materials and methods

### 2.1 Identification of WAK genes in the soybean genome

The genome data of soybean (glyma.Wm82.a4.v1) was downloaded from the Ensembl Plants database (http://ensemblgenomes.org/). The *AtWAK* genes of *Arabidopsis* were downloaded from the TAIR database (https://www.arabidopsis.org/) (Verica and He, 2002). The *At*WAK proteins were the query sequence, which blasted with the soybean genome in TBtools-II software. A total of 852 sequences were aligned at the threshold value 1e^−5^. Fifty-nine potential WAK genes were identified in the soybean genome based on the Swiss-Prot database.

The WAK domains, such as GUB_WAK_bind (PF13947), EGF (PF07645), and Pkinase (PF00069), were downloaded from the Pfam database (http://pfam.xfam.org/). The WAK sequences were used as the query in TBtools-II software, and an HMMER search was performed to identify *WAK* genes in the soybean genome (Chen et al., 2023). *WAK* genes were identified based on a threshold value (e^−3^). A total of 7, 100, and 2,231 genes were identified containing EGF, GUB_WAK_bind, or the Pkinase domain, respectively. There were 69 genes, including two types of domains: GUB_WAK_bind or EGF and Pkinase.

Ninety-four potential *WAK* genes were identified in the soybean genome through blast and HMMER searches. Further screening for the potential *WAK* genes was conducted in the Pfam (http://pfam.xfam.org/), SMART (http://smart.embl-heidelberg.de/), and NCBI CDD database (http://www.ncbi.nlm.nih.gov/cdd). Finally, 74 *WAK* genes were confirmed in the soybean genome, which contained at least two types of domains: GUB_WAK_bind or EGF and Pkinase domains.

The length and position information for 74 *GmWAK* genes were obtained from the gene annotations of the soybean genome and visualized using MapChart (Voorrips, 2002). The molecular weight (MW), isoelectric point (IP), instability index, and grand average of hydropathicity (GRAVY) were predicted with the Protein Parameter Calc in TBtools-II (Chen et al., 2023). The exon–intron structure was analyzed by GXF Stat in TBtools-II. The subcellular location of *GmWAK* genes was predicted using the online software DeepLoc-2.0 (https://services.healthtech.dtu.dk/services/DeepLoc-2.0/) (Almagro et al., 2017).

### 2.2 Gene phylogenetic, structure, and conserved motif analyses

The multiple sequences of *GmWAKs* and *AtWAKs/WAKLs* were aligned using the MUSCLE tool. The neighbor-joining method was used to build the phylogenetic tree using MEGA11 (Tamura et al., 2021). The parameters were set as follows: bootstrap method, Poisson model, 1,000 bootstrap replications, and partial deletion (Tamura et al., 2021). The phylogenetic tree was drawn on the website of EvolView v2 (He et al., 2016).

The Simple MEME Wrapper tool in TBtools-II was used to search for conserved motifs within *Gm*WAK proteins (Chen et al., 2023). The parameter settings were as shown below: the maximum number of motifs was 10, the motif length was 6–50 amino acids, the max E-value was e^−10^, and the model was any number of occurrences per seq. The conserved domain analysis of *Gm*WAKs was performed using the Batch SMART tool in TBtools-II (Chen et al., 2023). Visualizing the phylogenetic tree, gene structure, conserved motifs, and domains for *GmWAKs* was made within the Gene Structure View of TBtools-II (Chen et al., 2023).

### 2.3 Gene duplication and synteny analyses

Gene duplication (GD) was analyzed and visualized using TBtools-II (Chen et al., 2023). The simple Ka/Ks calculator was used to compute the non-synonymous substitution rate (Ka) and synonymous substitution rate (Ks) of duplicated gene pairs to evaluate GD events (Chen et al., 2023). The occurrence time of GD events was calculated using the following formula: *T* = (Ks/2*λ*) × 10^−6^, where *λ* = 6.161029 × 10^−9^ (Lynch and Conery, 2000). The syntenic relationships of different species were analyzed and visualized with One Step MCScanX and Multiple Synteny Plot in TBtools-II.

### 2.4 Cis-acting regulatory element analysis

The promoter sequence of the *GmWAK* genes was downloaded from the soybean genome, which covered the 2,000 bp upstream of the transcriptional start site. The cis-acting elements analysis was conducted using Plant CARE (http://bioinformatics.psb.ugent.be/webtools/plantcare/html/). The visualization of cis-acting elements was executed in TBtools-II and Adobe Photoshop 2023 (Chen et al., 2023).

### 2.5 Tissue-specific expression patterns of *GmWAK* genes

The RNA-Seq datasets of soybeans were obtained from the NCBI SRA database (http://www.ncbi.nlm.nih.gov/sra), including the gene expression information of different tissues at different developmental stages. The expression heat map of the *GmWAK* genes was drawn in TBtools-II.

### 2.6 Plant growth and stress treatment of soybean seedlings

The cultivated soybean variety Williams82 was employed to examine the gene profiles in response to stress treatments. Soybean seeds were sown in a double layer of absorbent paper and transferred to a half-strength Hoagland solution after 3 days. The conditions of the artificial illumination incubator were as follows: photoperiod 16 h light/8 h dark and temperature 25°C/18°C (day/night). After 10 days of cultivation, the seedlings were treated with multiple stress stimuli, such as cold (4°C), heat (40°C), salt (150 mM NaCl), drought (20% w/v PEG-6000), Al (100 μM of AlCl_3_), and Cd (100 μM of CdCl_2_). The root and leaves of seedlings were collected at 0, 3, 6, 12, and 24 h after treatment. Three replicates of three seedlings were collected for each treatment. All samples were rapidly frozen in liquid nitrogen and stored at −80°C for further analysis.

### 2.7 qRT-PCR analysis

The total RNA of the samples was extracted using a Total RNA Isolation Kit (Vazyme, RC411, China). The first strand of cDNA was synthesized using a reverse transcription kit (Vazyme, R312, China) according to the instructions. Quantitative real-time polymerase chain reaction (qRT-PCR) was performed on a LightCycler^®^ 480 system (Roche, Basel, Switzerland). The qRT-PCR system consisted of 2 µl of cDNA, 0.8 µl of primer (10 µμ), 7.2 µl of ddH_2_O, and 10 µl of SYBR qPCR master mix (Cat. No. Q711, Vazyme, China). The qRT-PCR reaction program was 95°C for 30 s, 40 cycles at 95°C for 10 s and 60°C for 30 s, 95°C for 15 s, 60°C for 60 s, and 95°C for 15 s. Relative quantification of gene expression was calculated using the 2^−ΔΔCT^ method, and *GmGAPDH* was used as a reference gene in qRT-PCR analysis (Livak and Schmittgen, 2001; Huang et al., 2015). The significance test of the gene expression was assessed by Tukey’s pairwise comparison test in SPSS software. The data were painted into graphs using GraphPad prism9.5 (https://www.graphpad.com/). The sequences of the gene-specific primers used in this study are listed in **Supplementary Table S1**.

## 3 Results

### 3.1 Identification, chromosomal localization, and physicochemical property analysis of the *WAK* gene family in soybeans

**Figure 1:**
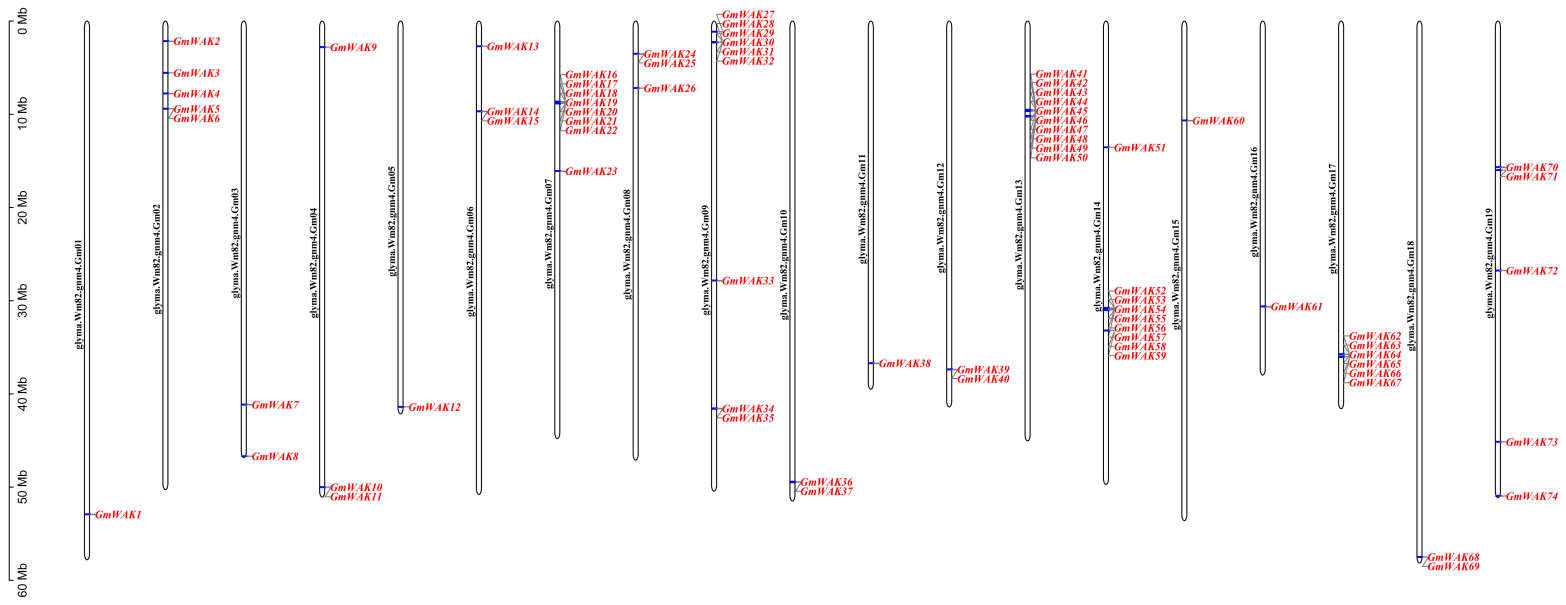
Chromosomal location of *GmWAK* genes in the soybean genome. Chromosome numbers are shown beside each chromosome. The scale on the left represents the chromosome length. The chromosome Gm 20 is not displayed, because no *WAK* gene was localized to that chromosome.

We identified 74 dependable *GmWAK* genes in the soybean genome using homology comparison and domain analysis. Sixty-one *GmWAK* genes contained GUB_WAK_bind or EGF and Pkinase conserved domains, and 13 genes contained GUB_WAK_bind, EGF, and Pkinase conserved domains. According to their positions on the chromosome, *GmWAK* genes were named *GmWAK1*–*GmWAK74* (**Figure 1**; **Supplementary Table S2**). Chromosomal mapping showed that the members of the *WAK* gene family were unevenly distributed on the 19 chromosomes, ranging from 1 to 10, with none on chromosome 20. The maximum number of *WAK* genes was located on Gm07, Gm09, Gm13, and Gm14 chromosomes containing 8, 9, 10, and 9 WAK genes, respectively. The length of the *Gm*WAK proteins was extremely varied. The shortest was *GmWAK60*, which encoded 476 amino acids, and the longest was *GmWAK39*, which encoded 987 amino acids. The molecular weight (MW) ranged from 53.60 kDa to 111.10 kDa. Among them, 48 *Gm*WAK proteins belonged to acidic proteins (PI < 7.0), and 26 *Gm*WAK proteins belonged to basic proteins (PI > 7.0). The instability index (InI) values ranged from 25.79 to 48.29. Thirty-two *Gm*WAK proteins with InI values were greater than 40, indicating that they might be more unstable than the other 42 *Gm*WAK proteins. The grand average of hydropathicity (GRAVY) index showed that 72 WAK proteins were hydrophilic (GRAVY < 0), and the rest were hydrophobic (GRAVY > 0). The subcellular location predicted that the *Gm*WAK proteins were localized to the cell membrane, and almost all contained signal peptides and transmembrane domains.

### 3.2 Phylogenetic, structural, and conserved motif characteristics of *GmWAK* genes

**Figure 2:**
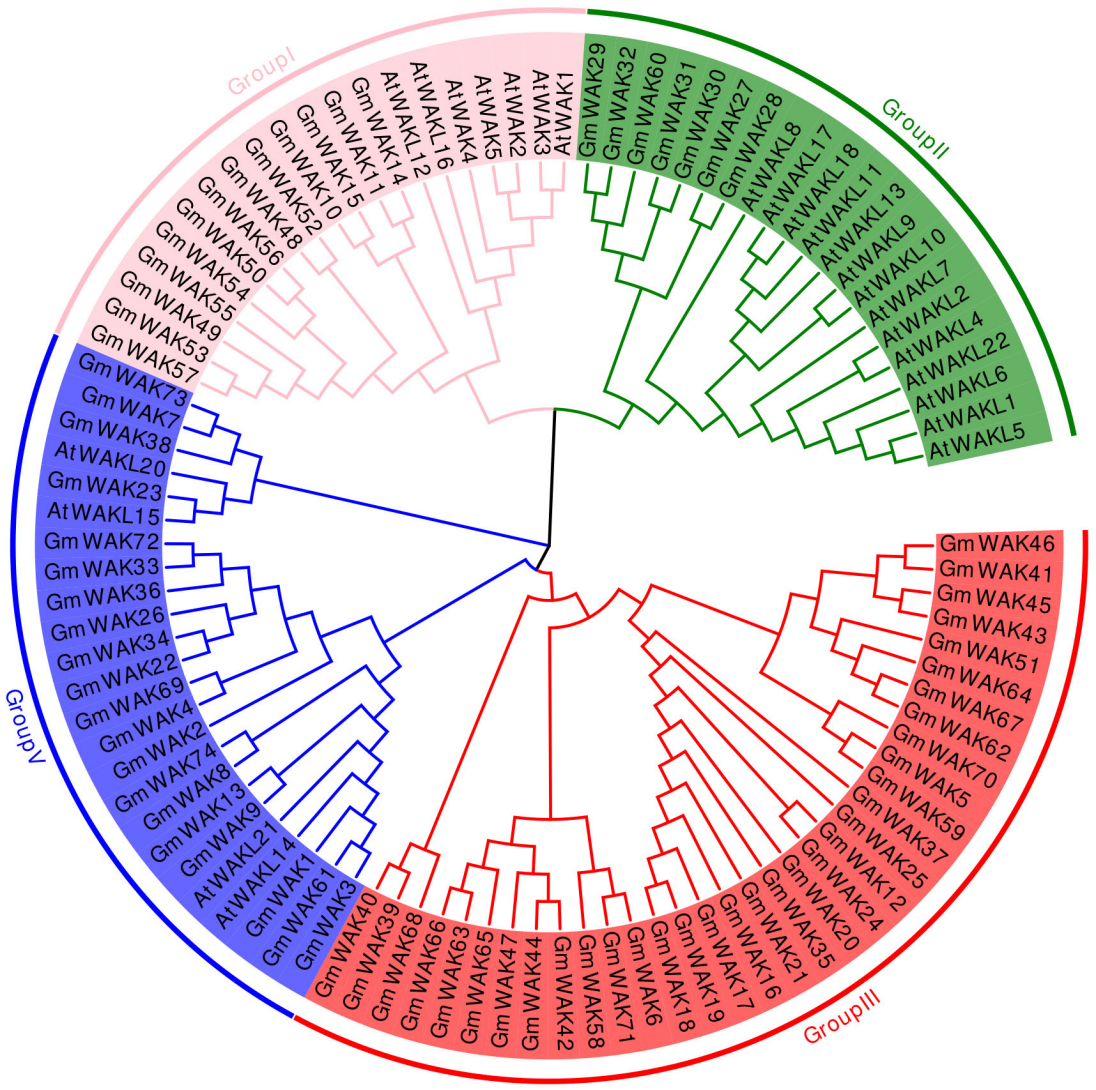
Phylogenetic tree analysis of the *WAK* gene family from *Arabidopsis* and soybean. Distinct color blocks represent different groups.

A phylogenetic tree was built using the neighbor-joining method with WAK proteins from soybean and *Arabidopsis* to explore the evolution of the *WAK* gene family. The *WAK* genes were divided into four groups: group I, group II, group III, and group IV (**Figure 2**). Group I clustered 13 *GmWAK* genes together with 7 *AtWAK/WAKL* genes. Group II included 7 *GmWAK* genes and 14 *AtWAKL* genes. Likewise, group IV contained 20 *GmWAK* genes and 4 *AtWAKL* genes. Group III was the largest one, containing 34 *GmWAK* genes without any member from *Arabidopsis* included within this group.

**Figure 3:**
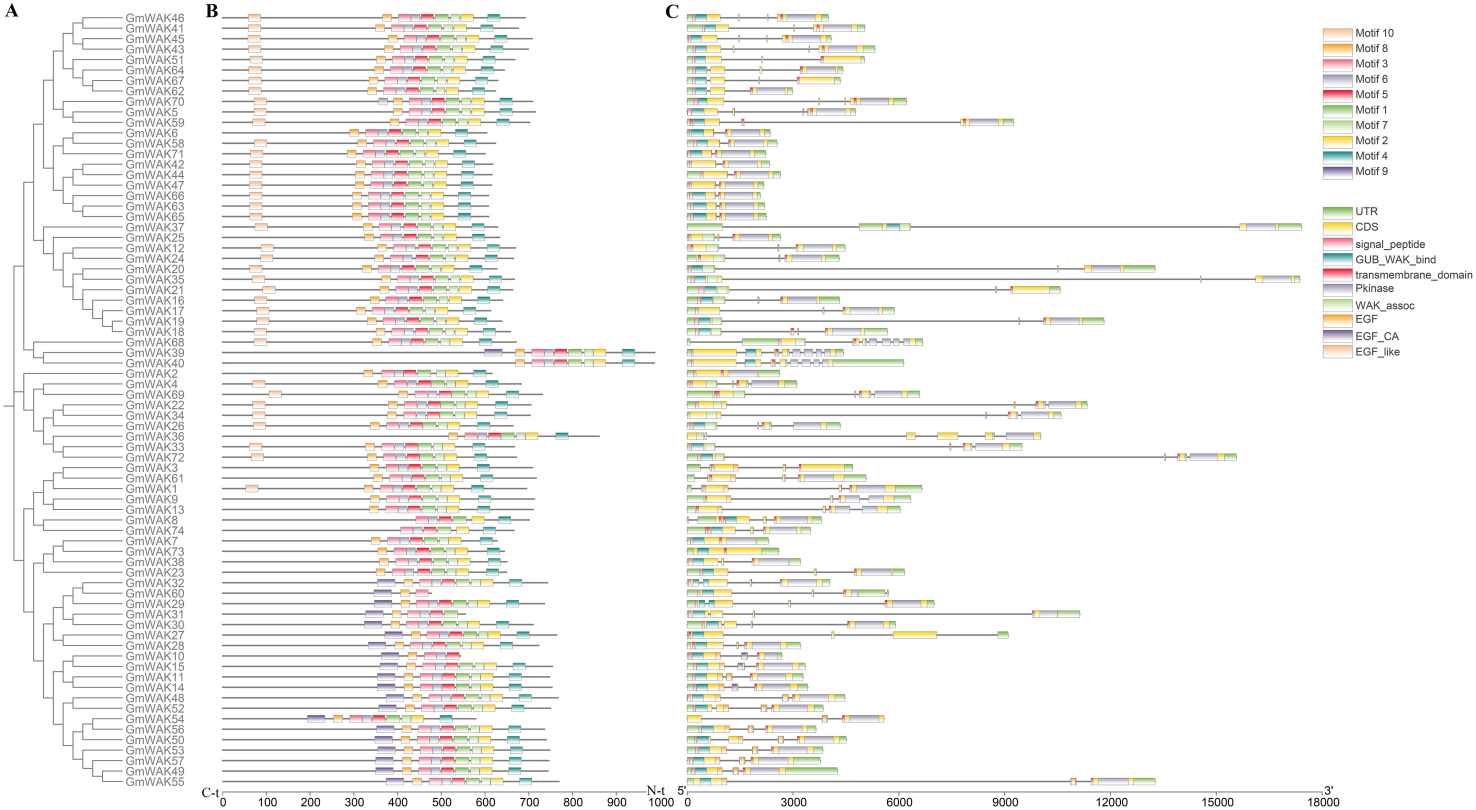
Gene structure analysis of *Gm*WAK proteins. **(A)** The phylogenetic tree of the 74 *Gm*WAK proteins. **(B)** The motif distribution of the 74 *Gm*WAK proteins. **(C)** The intron–exon structure and conservative domain of the *Gm*WAK protein sequences.

Based on gene structure analysis, one to eight exons were observed in the *GmWAK* genes, with a mean of 3.43 exons per gene (**Figure 3**; **Supplementary Table S2**). The proportion of *GmWAK* genes containing one, two, three, four, six, and eight exons was 4.05%, 12.16%, 39.18%, 39.18%, 1.35%, and 4.05%, respectively. The number of exons in groups I and II was three or four. In group III, we detected two to eight exons in *GmWAK* genes, and 41.17% of genes in group III were composed of three exons. A similar variation of exons was observed in group IV, and 65% of the *GmWAK* genes included four exons. We also found that the range of *GmWAK* gene lengths was from 2,068 to 20,003 bp, and the coding sequences (CDS) were 1,428 to 2,961 amino acid residues. There was an extensive length variation in *GmWAK* genes due to differences in the number and length of introns.

We analyzed the motif distribution of *Gm*WAK proteins, and 10 motifs were predicted (**Figure 3**; **Supplementary Tables S3**, **S4**). Similar motif distributions were observed among *Gm*WAK members. Approximately 93.2% (69) of *Gm*WAK proteins shared eight conserved motifs (motifs 1–8), and 6.75% (5) of *Gm*WAK proteins were observed to have two to six motifs from motifs 1 to 8. Furthermore, motif 9 was specific to group I and II members, and motif 10 was present in the *Gm*WAK proteins from groups III and IV (**Supplementary Table S3**).

According to the conserved domain analysis, the *Gm*WAK proteins contained a highly conserved domain structure. All *Gm*WAK proteins had the Pkinase and transmembrane domains at the C-terminal end, and 77.02% of *Gm*WAK proteins had signal peptides at the N-terminal end (**Supplementary Table S2**; **Figure 3**). The WAK proteins had the GUB_WAK_bind and EGF domains. All *Gm*WAK proteins contained the GUB_WAK_bind and EGF domains in group I, except *Gm*WAK54. In groups II and III, all *Gm*WAK proteins contained only the GUB_WAK_bind domain without the EGF domain. In group IV, 70% and 20% of the *Gm*WAK proteins included either the GUB_WAK_bind or the EGF-related domains, respectively. The remaining 10% of *Gm*WAK proteins simultaneously contained the two domains. The *Gm*WAK proteins in the same group displayed similar conserved domains and structures, indicating they might have comparable functions.

### 3.3 Duplication and synteny analysis of *GmWAK* genes

**Figure 4:**
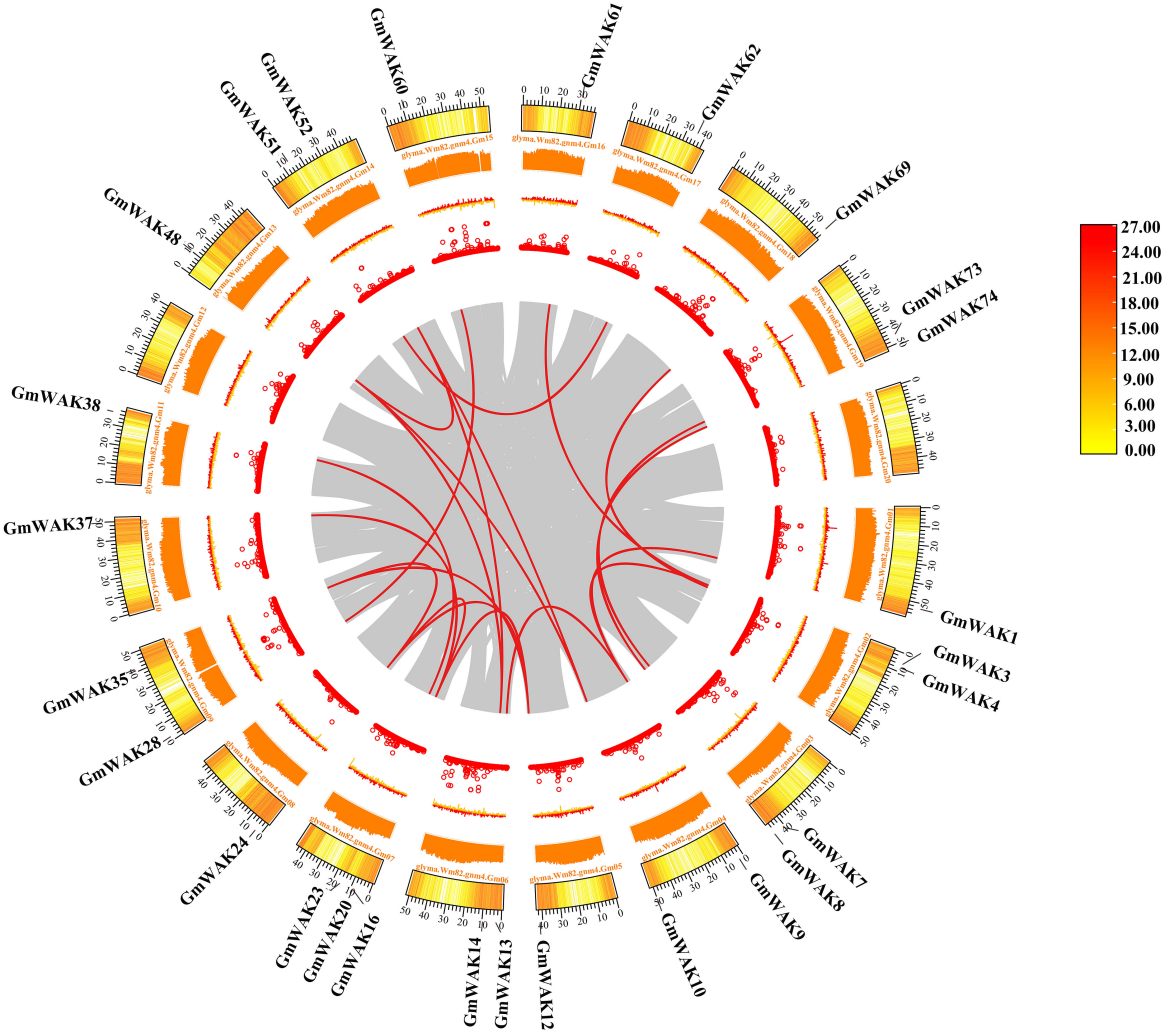
Synteny analysis of the *GmWAK* genes. The gray lines represent the collinear gene pairs in the whole soybean genome, and the red lines represent the collinear pair of *GmWAK* genes. The circles represent gene density, N ratio, GC skew, and GC ratio from inside to outside, respectively.

GD events, which are the main reason for the expansion of gene family members, are usually classified into segmental duplication (SD), tandem duplication (TD), and proximal duplication (PD). We conducted a GD analysis of the soybean *WAK* gene family. A total of 51 *GmWAK* genes participated in the expansion process of the *GmWAK* gene family, which included 21 pairs of SD, nine pairs of TD, and six pairs of PD (**Table 1**; **Figure 4**). The non-synonymous (Ka) and synonymous (Ks) substitution rates were calculated to investigate the selection pressures after GD. The Ka values ranged from 0.005 to 0.612, the Ks values ranged from 0.008 to 3.58, and the Ka/Ks values of 34 gene pairs ranged from 0.12 to 0.94, indicating that the *GmWAKs* evolved in purifying selection after GD (Ka/Ks < 1).

**Table 1:** Selective pressure analysis of *GmWAKs*.

Duplicated gene pairs	Ka	Ks	Ka/Ks	Duplicated type	Purifying selection	Time (Mya)
*GmWAK17/GmWAK18*	0.19	0.39	0.49	Tandem	Purifying selection	31.46
*GmWAK18/GmWAK19*	0.04	0.07	0.58	Tandem	Purifying selection	5.88
*GmWAK29/GmWAK30*	0.18	0.28	0.64	Tandem	Purifying selection	22.66
*GmWAK30/GmWAK31*	0.06	0.11	0.59	Tandem	Purifying selection	8.90
*GmWAK31/GmWAK32*	0.17	0.28	0.61	Tandem	Purifying selection	23.09
*GmWAK45/GmWAK46*	0.07	0.08	0.94	Tandem	Purifying selection	6.42
*GmWAK49/GmWAK50*	0.19	0.43	0.43	Tandem	Purifying selection	35.23
*GmWAK53/GmWAK54*	0.15	0.35	0.43	Tandem	Purifying selection	28.55
*GmWAK55/GmWAK56*	0.23	0.65	0.35	Tandem	Purifying selection	52.40
*GmWAK41/GmWAK42*	0.58	2.55	0.23	Proximal	Purifying selection	207.14
*GmWAK43/GmWAK44*	0.61	2.37	0.26	Proximal	Purifying selection	191.98
*GmWAK63/GmWAK64*	0.59	2.17	0.27	Proximal	Purifying selection	176.51
*GmWAK64/GmWAK65*	0.59	2.12	0.28	Proximal	Purifying selection	172.29
*GmWAK65/GmWAK66*	0.00	0.01	0.66	Proximal	Purifying selection	0.61
*GmWAK66/GmWAK67*	0.59	2.31	0.26	Proximal	Purifying selection	187.82
*GmWAK1/GmWAK9*	0.34	2.64	0.13	Segmental	Purifying selection	213.92
*GmWAK3/GmWAK9*	0.37	1.78	0.21	Segmental	Purifying selection	144.79
*GmWAK3/GmWAK61*	0.04	0.16	0.23	Segmental	Purifying selection	13.10
*GmWAK4/GmWAK69*	0.22	0.43	0.51	Segmental	Purifying selection	34.60
*GmWAK7/GmWAK73*	0.02	0.13	0.19	Segmental	Purifying selection	10.25
*GmWAK8/GmWAK74*	0.04	0.14	0.31	Segmental	Purifying selection	11.24
*GmWAK9/GmWAK13*	0.04	0.18	0.22	Segmental	Purifying selection	14.56
*GmWAK10/GmWAK48*	0.53	NA	NA	Segmental	NA	NA
*GmWAK10/GmWAK52*	0.59	2.79	0.21	Segmental	Purifying selection	226.81
*GmWAK12/GmWAK16*	0.33	1.12	0.29	Segmental	Purifying selection	90.99
*GmWAK12/GmWAK24*	0.05	0.13	0.36	Segmental	Purifying selection	10.35
*GmWAK12/GmWAK35*	0.36	1.53	0.24	Segmental	Purifying selection	124.16
*GmWAK14/GmWAK48*	0.42	1.99	0.21	Segmental	Purifying selection	161.60
*GmWAK14/GmWAK52*	0.44	2.23	0.20	Segmental	Purifying selection	181.06
*GmWAK16/GmWAK24*	0.33	1.19	0.28	Segmental	Purifying selection	96.91
*GmWAK20/GmWAK37*	0.44	3.58	0.12	Segmental	Purifying selection	290.33
*GmWAK23/GmWAK38*	0.41	NA	NA	Segmental	NA	NA
*GmWAK24/GmWAK35*	0.38	1.69	0.22	Segmental	Purifying selection	137.10
*GmWAK28/GmWAK60*	0.34	0.86	0.40	Segmental	Purifying selection	69.49
*GmWAK48/GmWAK52*	0.20	0.60	0.33	Segmental	Purifying selection	48.40
*GmWAK51/GmWAK62*	0.11	0.31	0.35	Segmental	Purifying selection	25.28

Ka and Ks indicate the non-synonymous and synonymous were used to determine the selective pressure after duplication. Ka/Ks = 1 indicates the neutral selection, Ka/Ks > 1 indicates the positive selection, and Ka/Ks < 1 indicates the purifying selection. The duplication date (Million years ago, Mya) was calculated by the formula: *T* = (Ks/2*λ*) × 10^−6^, where *λ* = 6.161029 × 10^−9^. NA indicates that it is unable to calculate Ka/Ks values.

**Figure 5:**
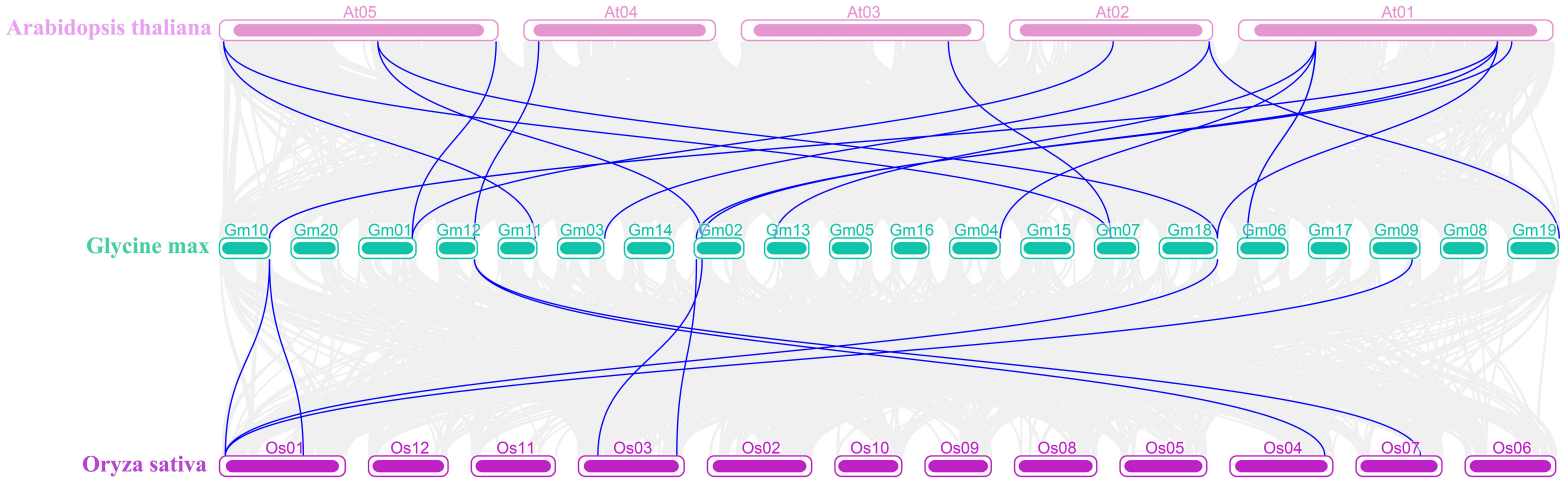
Synteny analysis of soybean with *Arabidopsis* and rice. The gray lines represent the collinear gene pairs of soybean with *Arabidopsis* and rice in the whole genome, and the blue lines represent the collinear pairs of the WAK gene family from those three species.

Additionally, Ks was used to estimate the divergence time in evolution. GD events occurred between 0.61 Mya and 290.33 Mya, averaging 91.23 Mya. We performed the synteny analysis using MCScanX in TBtools-II to further explore the evolutionary relationship of the *WAK* gene family in different species. The comparison of soybeans with *Arabidopsis* and rice at the genome level showed that 31,623 collinear pairs were observed between soybeans and *Arabidopsis*, and 10,953 collinear pairs were observed between soybeans and rice, among which the number of collinear pairs belonging to the WAK genes were eight and two, respectively (**Figure 5**; **Supplementary Tables S5**, **S6**).

### 3.4 Cis-acting element analysis of the *GmWAK* genes

**Figure 6:**
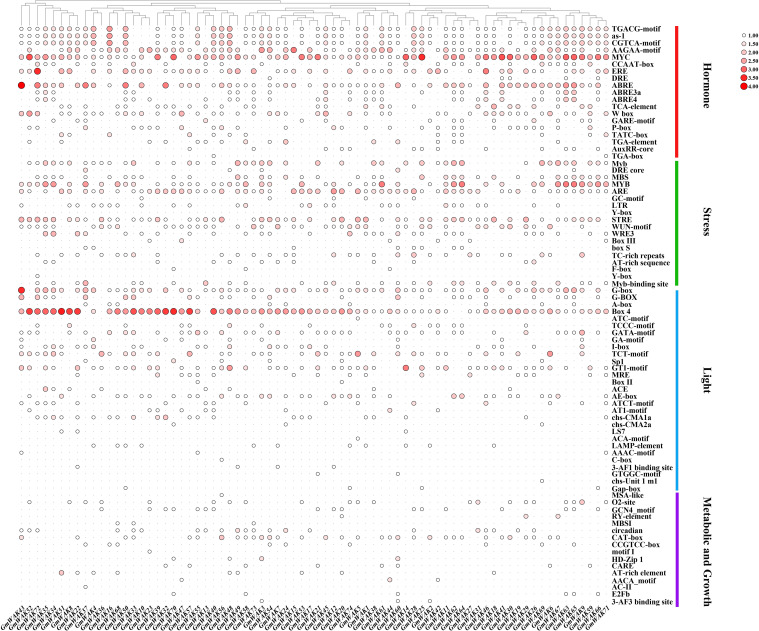
Cis-acting elements in the promoter regions of *GmWAK* genes.

After cis-acting analysis of the *GmWAK* genes, 96 types of elements were identified in promoter regions, which were divided into six subgroups: core promoter elements, hormone-responsive elements, stress-responsive elements, light-responsive elements, metabolic- and growth-related elements, and unknown function elements (**Figure 6**; **Supplementary Tables S7**, **S8**). The core promoter elements (e.g., CAAT-box, TATA-box, AT~TATA-box) were identified in the promoter regions of all *GmWAK* genes. There were 19 types of hormone-responsive elements, which included MJA and JA (4), ABA (4), ETH (3), GA (3), IAA (3), and SA (2). Among the hormone-responsive elements, MJA- and JA-responsive elements occurred most frequently at 412 times and were distributed on the promoter regions of all *GmWAK* genes except *GmWAK33.* ETH-responsive elements were identified in promoter regions of *GmWAK* genes, where MYC- and ERE-responsive elements appeared 365 times. ABA and SA responsive elements were separately found in 81.08% and 71.62% of the *GmWAK* genes. Among them, the elements ABRE and W box were the most frequent. GA- and IAA-responsive elements were identified in 58.1% and 40.50% of the *GmWAK* genes. A total of 18 types of stress-responsive elements were found in the *GmWAK* genes. The drought-related elements MYB, Myb, and MBS were the most frequent, followed by the anaerobic-responsive elements (ARE), heat-responsive elements (STRE), and trauma-responsive elements (WUN-motif, WRE3). Approximately 29 light-responsive elements were near the *GmWAK* genes, among which Box 4 and G-box occurred in 98% of the *GmWAK* genes. Sixteen different elements were associated with the metabolism and growth of the plant. CAT-box, O^2^-site, and circadian were the most frequent elements related to meristem, gliadin metabolism, and circadian regulation. Furthermore, 11 unknown functional elements were predicted in the soybean *WAK* gene family.

### 3.5 Tissue expression profile analysis of the soybean *WAK* gene family

**Figure 7:**
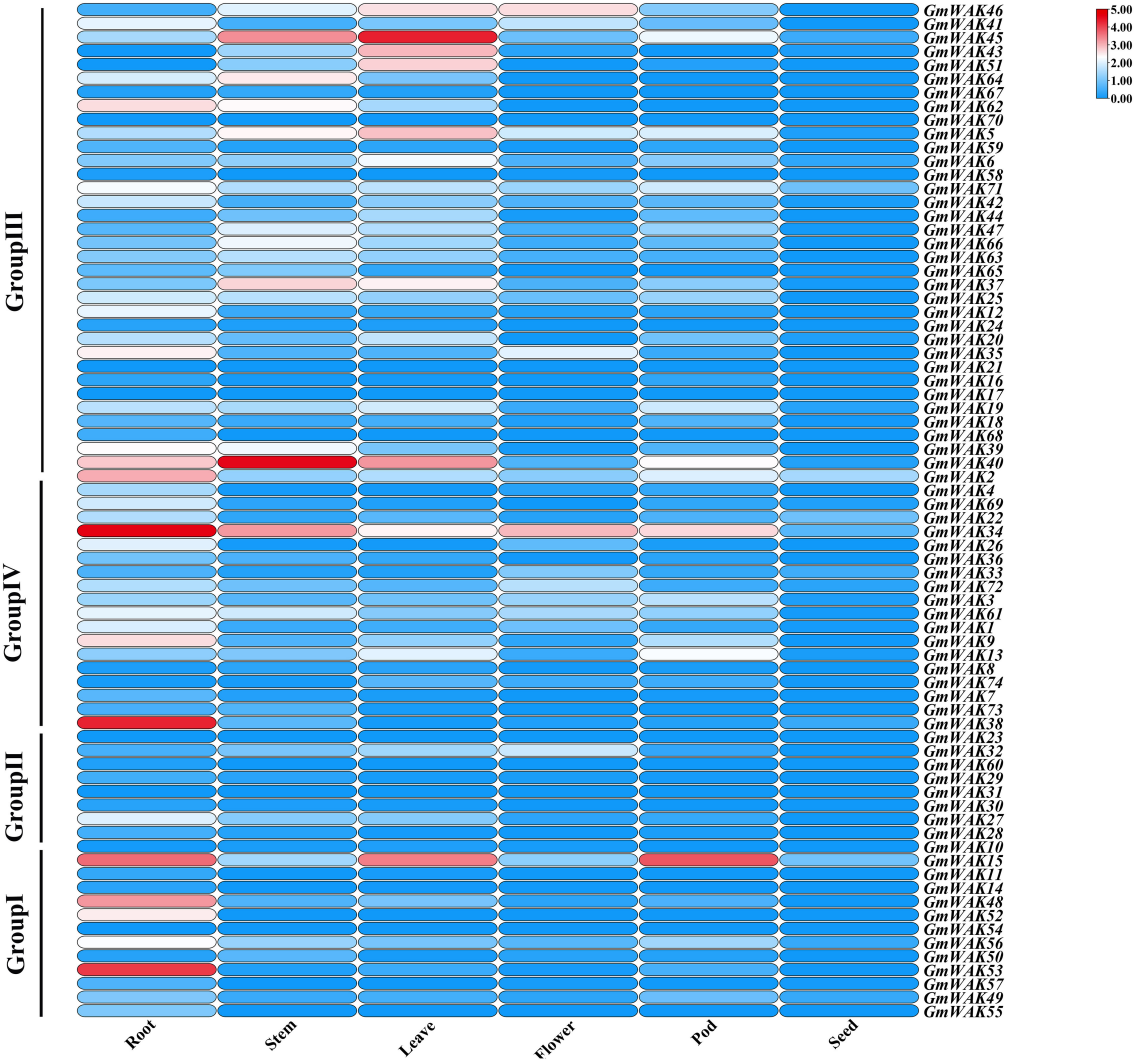
The relative expression of 74 *GmWAK* genes in soybean tissues.

We obtained the RNA-Seq data of seven tissues from the NCBI database (https://www.ncbi.nlm.nih.gov/sra). We conducted a tissue expression analysis of 74 *GmWAK* genes (**Figure 7**; **Supplementary Table S9**) to explore the putative function of *GmWAK* genes in soybean development. Forty-seven *GmWAK* genes were expressed in soybean tissues, while 25 genes were almost not expressed by its fragments per kilobase of exon per million fragments mapped (FPKM) values. There were 1, 13, 18, 26, 29, and 38 *GmWAK* genes expressed in the seed, flower, pod, stem, leaf, and root, respectively (FPKM > 1). Among these, six *GmWAK* genes showed high expression (FPKM > 10), such as *GmWAK15* in the root, leaf, and pod; *GmWAK34*, *GmWAK38*, and *GmWAK53* in the root; and *GmWAK40* and *GmWAK45* in the stem or leaf.

### 3.6 Expression profile analysis of *GmWAK* genes under different abiotic stresses

We treated soybean seedlings with various abiotic stresses, including salt (150 mM of NaCl), drought (20% PEG6000), cold (4°C), heat (40°C), Al (100 µM of AlCl_3_), and Cd (100 µM of CdCl_2_) to further validate the response pattern of the *GmWAK* genes. Then, we detected the expression of 18 *GmWAK* genes using the qRT-PCR method.

#### 3.6.1 Responses of *GmWAK* genes to cold stress

**Figure 8:**
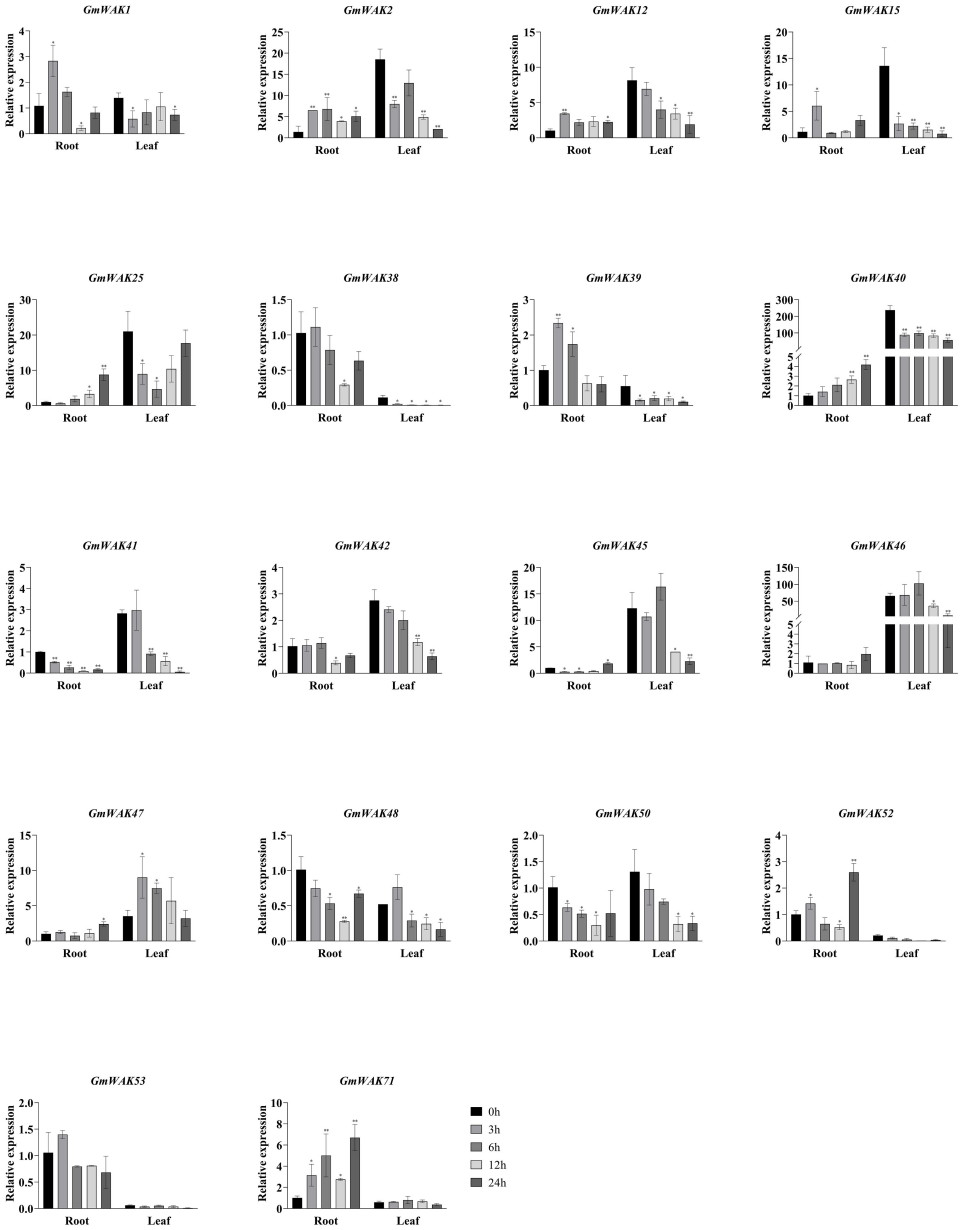
The expression levels of 18 *GmWAK* genes in soybean seedlings after cold (4°C) treatment. The abscissa indicates the time points of the leaf and root after the stress treatments. Data represent the average of three independent biological replicates ± SD. “*” and “**” indicate “*P* < 0.05” and “*P* < 0.01,” respectively, and mean significant difference occurred after treatment.

Seventeen differentially expressed *GmWAK* genes were identified under cold stress. The expression of these genes in cold conditions changed more than two-fold than that in the control group (**Figure 8**). A total of 10 *GmWAK* genes were significantly upregulated in soybean roots. Among them, *GmWAK1/2/12/15/39* peaked after 3 h of cold treatment and were 2.3–5.3 times the control. *GmWAK25/40/47/52/71* reached the maximum after 24 h and were 2.4–8.6 times the control. Five *GmWAK* genes were significantly downregulated in soybean roots. *GmWAK38/41/42/48/50* decreased to 9.8%–38.2% of the control after 12 h of treatment. The expression level of *GmWAK47* in soybean leaves was up to 2.6 times that of the control at 3 h after treatment. Fourteen significantly downregulated *GmWAK* genes were identified. *GmWAK1* was 41.3% of the control after 3 h of treatment, and *GmWAK25* was 22.2% of the control after 6 h. The remaining 12 *GmWAK* genes were reduced to 2.2%–52.8% of the control group after 24 h of treatment. Among the 17 significantly differentially expressed *GmWAK* genes, the relative expression levels of *GmWAK2/15/25/40/45/46* were high, which were significantly increased by 4.1–8.6 times in the root and yet were significantly decreased by 71.5%–94.4% in the leaves.

#### 3.6.2 Responses of *GmWAK* genes to heat stress

**Figure 9:**
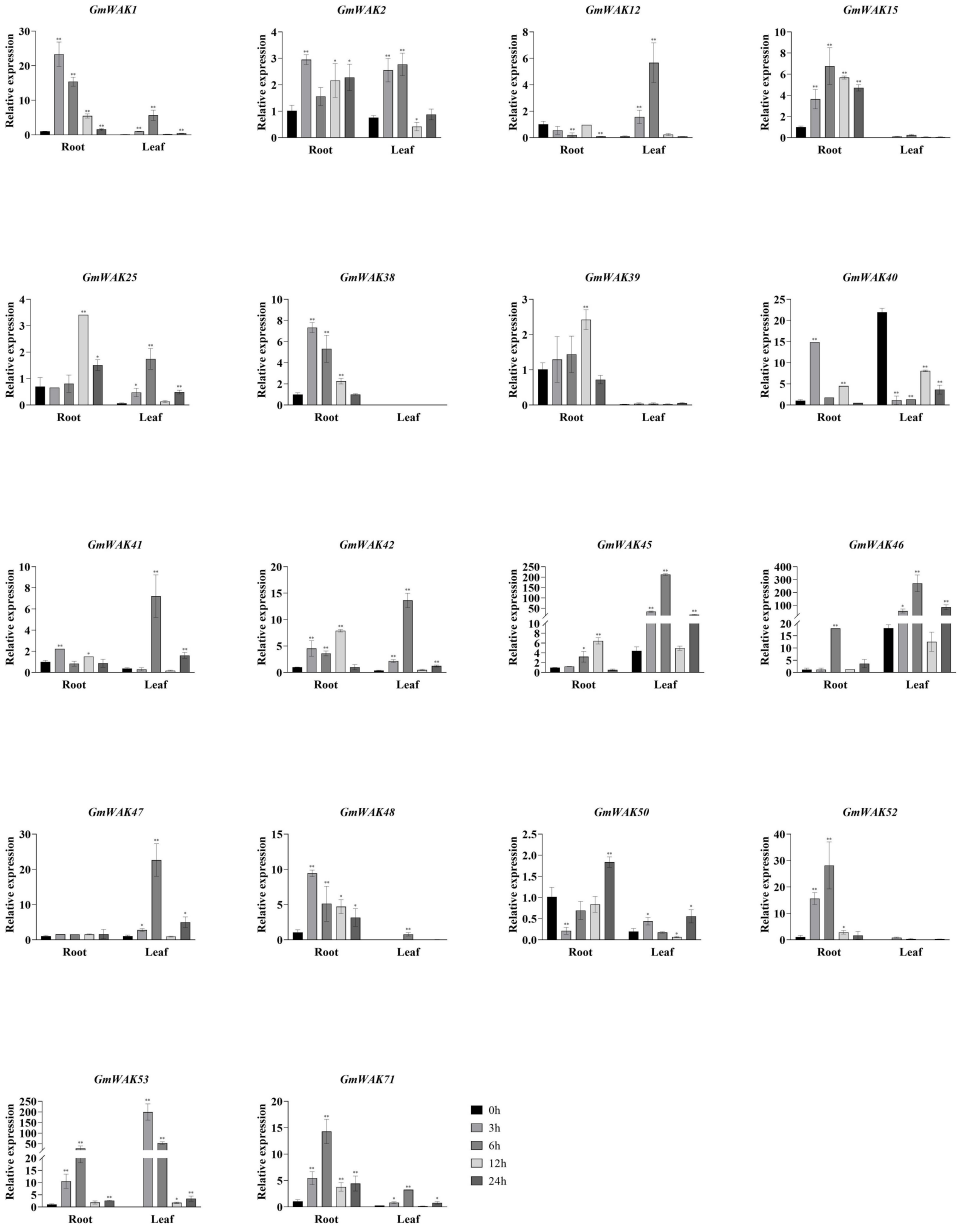
The expression levels of 18 *GmWAK* genes in soybean seedlings after heat (40°C) treatment. The abscissa indicates the time points of the leaf and root after the stress treatments. Data represent the average of three independent biological replicates ± SD. “*” and “**” indicate “*P* < 0.05” and “*P* < 0.01,” respectively, and mean significant difference occurred after treatment.

Eighteen differentially expressed *GmWAK* genes were identified in soybeans under heat stress, which changed more than two-fold than that in the control group (**Figure 9**). Fifteen significantly upregulated *GmWAK* genes were detected in soybean roots under heat stress, *GmWAK1/2/38/40/41/48* were up to 2.2–23.3 times the control at 3 h, *GmWAK15/46/52/53/71* were up to 6.7–28.3 times the control at 6 h, and *GmWAK25/39/42/45* were up to 2.4–7.9 times the control at 12 h. One *GmWAK* gene showed an expression pattern of first decreasing and then increasing. *GmWAK50* significantly decreased by 79.2% after 3 h of treatment and increased by 82.0% after 24 h. Eleven differentially expressed *GmWAK* genes were identified under heat stress in soybean leaves. The expression level of *GmWAK53* was sharply increased to 43,506 times that in the control after 3 h of treatment. *GmWAK1/2/12/25/41/42/45/46/47/71* increased to 3.7–57.3 times the control group after 6 h of treatment. One *GmWAK* gene was significantly downregulated under heat stress. *GmWAK40* was reduced to 6.2% of the control group after 6 h of treatment. Among the 18 significantly differentially expressed *GmWAK* genes, there were nine *GmWAK* genes with high expression levels, *GmWAK1/42/45/46/47/52/53/71* were significantly increased by 6.4–43506 times in heat stress conditions, and *GmWAK40* was upregulated by 14.5 times in the roots and yet was decreased by 93.8% in the leaves.

#### 3.6.3 Responses of *GmWAK* genes to drought stress

**Figure 10:**
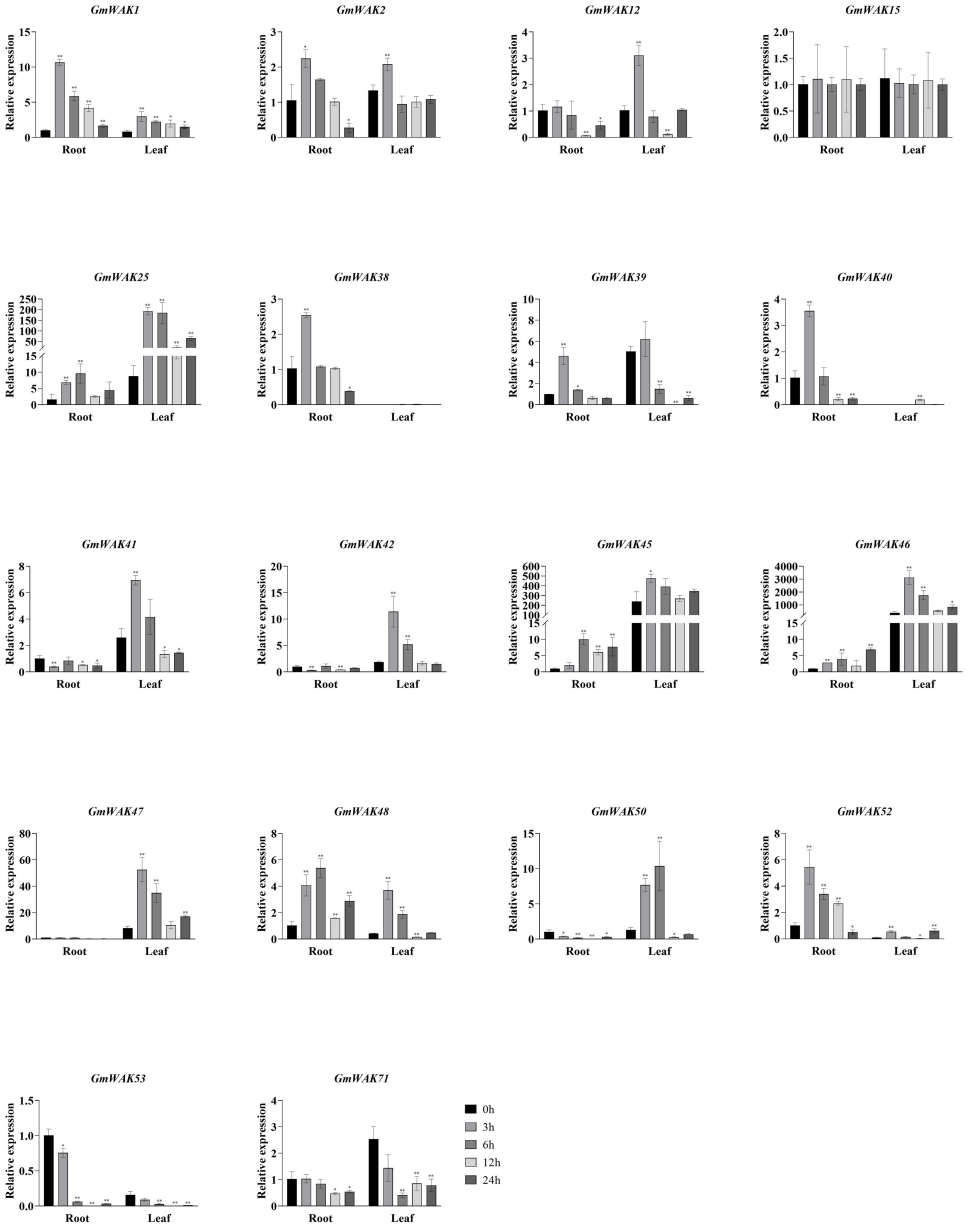
The expression levels of 18 *GmWAK* genes in soybean seedlings after drought treatment. The abscissa indicates the time points of the leaf and root after the stress treatments. Data represent the average of three independent biological replicates ± SD. “*” and “**” indicate “*P* < 0.05” and “*P* < 0.01,” respectively, and mean significant difference occurred after treatment.

Seventeen differentially expressed *GmWAK* genes were identified in soybeans under drought stress. These genes changed more than two-fold than those in the control group (**Figure 10**). There were 10 *GmWAK* genes significantly upregulated under drought stress in soybean roots. The expression levels of *GmWAK1/2/38/39/40/52* reached their maximum at 3 h after treatment and were 2.1–10.6 times that of the control. *GmWAK25/45/48* increased to 5.2–9.9 times the control at 6 h. *GmWAK46* increased to 6.9 times the control at 24 h. Six *GmWAK* genes were significantly downregulated in soybean roots. The expression levels of *GmWAK41/42* were reduced by 61.4% and 70% after 3 h of treatment, and *GmWAK12/50/53/71* were reduced by 53.8%–99.7% at 12 h after treatment. Eleven *GmWAK* genes showed a significant increase under drought stress in soybean leaves. The expression of *GmWAK1/2/12/25/41/42/45/46/47/48* increased to 1.6–21.9 times that of the control at 3 h after treatment. *GmWAK50* was upregulated 8.1 times over the control at 6 h. Two *GmWAK* genes showed a significant decrease under drought stress, and the expression of *GmWAK71* and *GmWAK39* decreased by 83.7% and 99.7% at 6 h and 12 h, respectively. Five *GmWAK* genes had high expression levels among the 17 significantly differentially expressed genes. *GmWAK25/45/46/47* were significantly increased by 2.0–21.9 times under heat stress, and *GmWAK42* was downregulated by 70.0% in soybean roots and increased by 6.1 times in soybean leaves.

#### 3.6.4 Responses of *GmWAK* genes to salt stress

**Figure 11:**
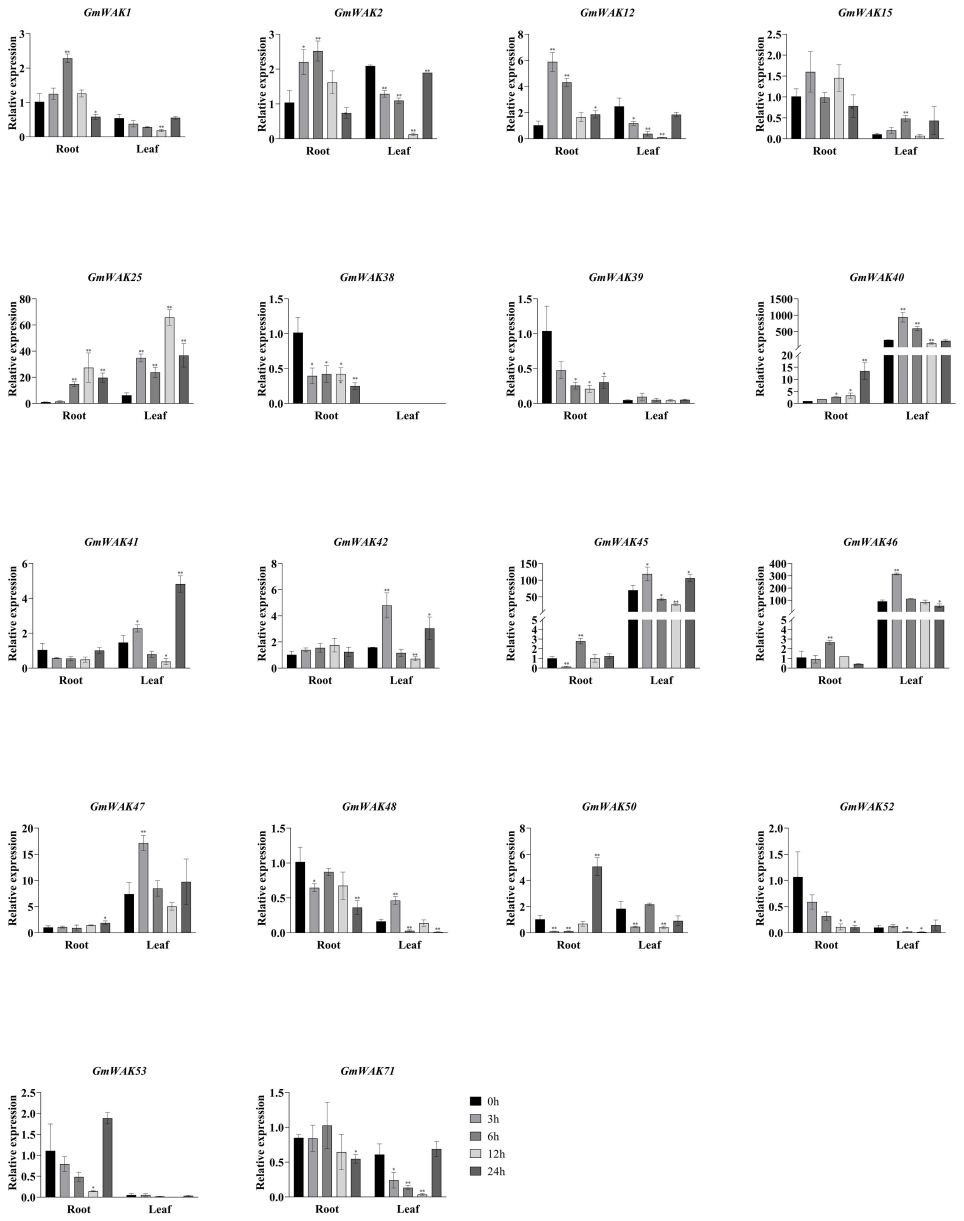
The expression levels of 18 *GmWAK* genes in soybean seedlings after salt treatment. The abscissa indicates the time points of the leaf and root after the stress treatments. Data represent the average of three independent biological replicates ± SD. “*” and “**” indicate “*P* < 0.05” and “*P* < 0.01,” respectively, and mean significant difference occurred after treatment.

There were 18 differentially expressed *GmWAK* genes identified in soybeans, whose expression level was more than twice that of the control under salt stress (**Figure 11**). Eight *GmWAK* genes showed a significant increase in soybean roots under high salt treatment. The expression of *GmWAK12* increased to 5.7 times the control at 3 h after treatment, *GmWAK1/2/45/46* were up to 2.3–2.8 times the control at 6 h, *GmWAK25* achieved 27.4 times of the control at 12 h, and *GmWAK40/50* increased by 4.9 and 13.4 times at 24 h, respectively. Six *GmWAK* genes showed a significant decrease under high-salt treatment. *GmWAK39/53* decreased by 80% and 87.2% at 12 h after treatment. *GmWAK38/48/52/71* decreased by 35.6%–90.2% at 24 h. Nine *GmWAK* genes significantly increased under high salt treatment in soybean leaves. The expression of *GmWAK40/42/45/46/47/48* increased by 1.7–3.9 times that of the control at 3 h after treatment. *GmWAK15*, *GmWAK 25*, and *GmWAK 41* increased by 4.5, 10.7, and 3.3 times at 6 h, 12 h, and 24 h, respectively. Five *GmWAK* genes showed a significant decrease in soybean leaves. The expression of *GmWAK1/2/12/50/71* was reduced to 4.6%–34.1% of the control group at 12 h after treatment. Among the 18 significantly differentially expressed genes, the relative expression of *GmWAK25/40/45/46/47* was at high levels, significantly increasing by 2.4–27.4 times and 1.7–10.7 times in the roots and leaves, respectively.

#### 3.6.5 Responses of *GmWAK* genes to Al stress

**Figure 12:**
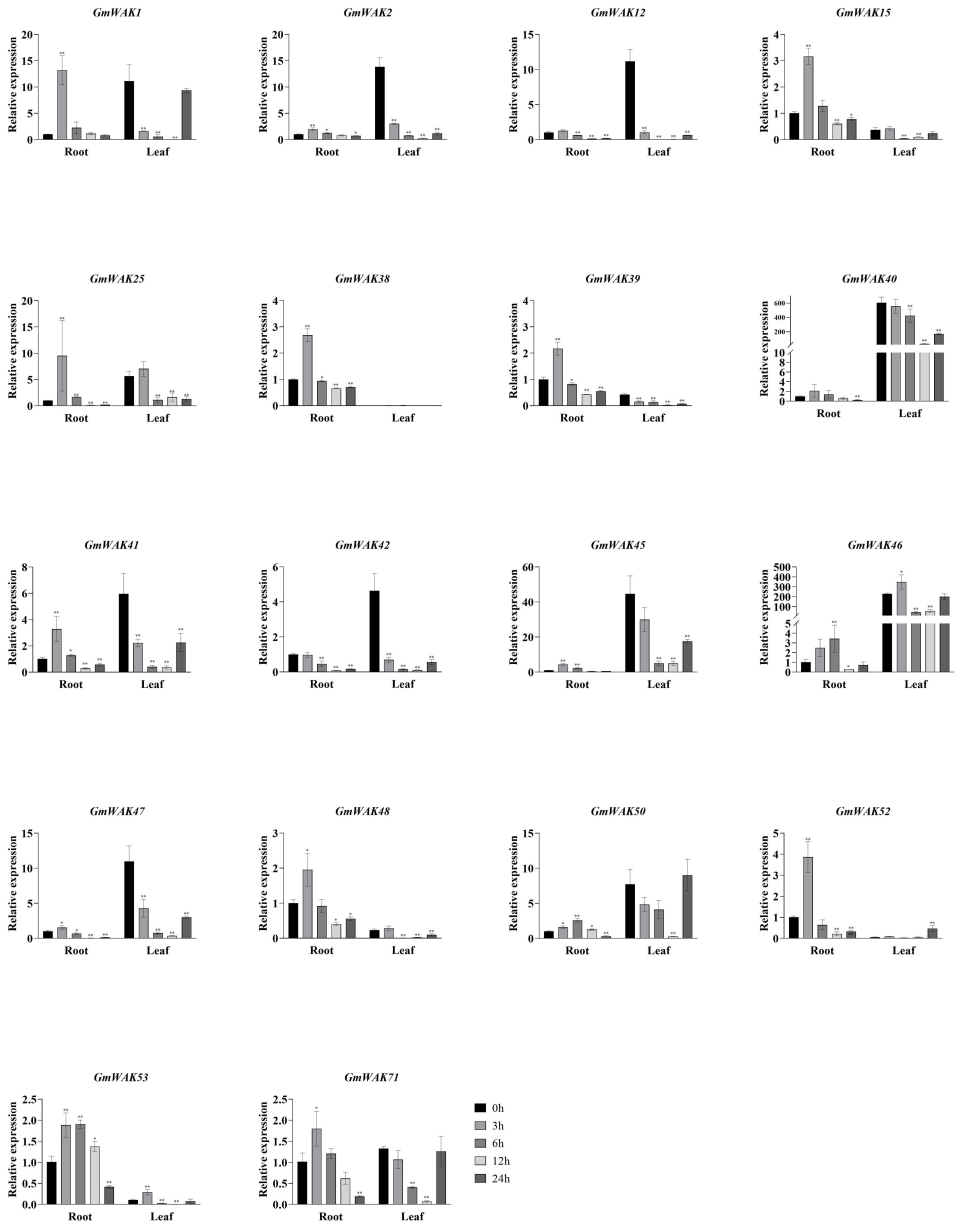
The expression levels of 18 *GmWAK* genes in soybean seedlings after Al treatment. The abscissa indicates the time points of the leaf and root after the stress treatments. Data represent the average of three independent biological replicates ± SD. “*” and “**” indicate “*P* < 0.05” and “*P* < 0.01,” respectively, and mean significant difference occurred after treatment.

Eighteen differentially expressed *GmWAK* genes were observed in soybeans under Al stress. These genes changed more than two-fold than those in the control group (**Figure 12**). Fifteen *GmWAK* genes in soybean roots were significantly upregulated under Al stress. The expression of *GmWAK46/50* increased 3.4 and 2.5 times, respectively, at 6 h after treatment. The remaining 13 *GmWAK* genes increased 1.5–13.2 times after 3 h of treatment. Three *GmWAK* genes were significantly downregulated in soybean roots. The expression of *GmWAK12/42* was reduced by 87.4% and 91.9% at 12 h after treatment, and *GmWAK40* was decreased by 73.7% at 24 h. Three *GmWAK* genes in soybean leaves were significantly upregulated by Al stress. The expression of *GmWAK46/53* was increased to 1.5 and 2.8 times that of the control at 3 h after treatment. *GmWAK52* was increased eight times at 24 h. Fifteen *GmWAK* genes were significantly downregulated in soybean leaves. *GmWAK12/15/25/46/48* expression was reduced by 79.8%–99.8% at 6 h after treatment. *GmWAK1/2/39/40/41/42/45/47/50/71* reduced by 89.0%–99.3% at 12 h. Among the 18 significantly differentially expressed genes, seven *GmWAK* genes had high expression levels in soybeans. The expression of *GmWAK1/2/45/46/47* was significantly increased by 1.5–13.2 times in the roots but decreased by 81.7%–99.7% in the leaves. *GmWAK12/40* decreased by 73.7%–99.8% in soybean roots and leaves.

#### 3.6.6 Responses of *GmWAK* genes to Cd stress

**Figure 13:**
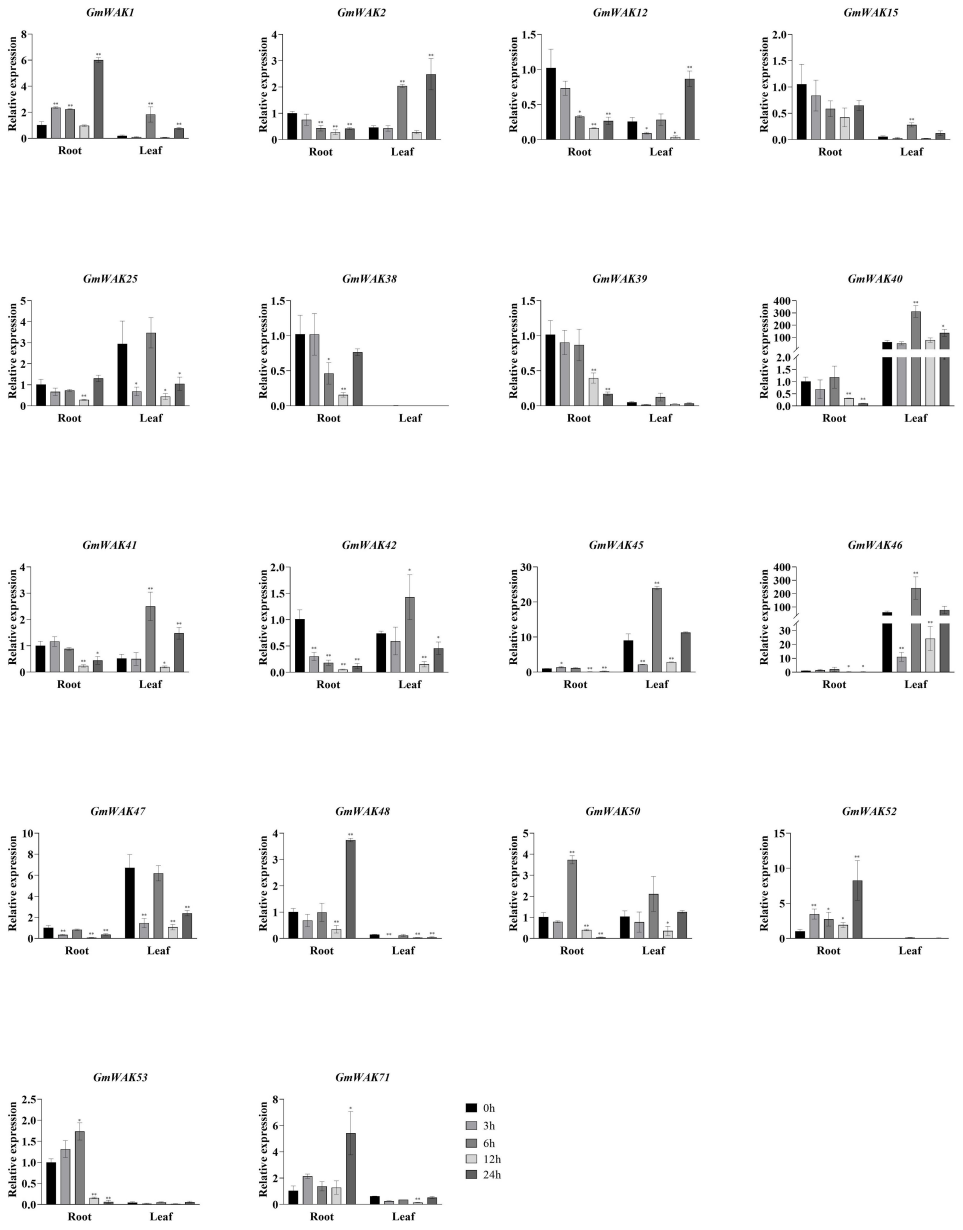
The expression levels of 18 *GmWAK* genes in soybean seedlings after Cd treatment. The abscissa indicates the time points of the leaf and root after the stress treatments. Data represent the average of three independent biological replicates ± SD. “*” and “**” indicate “*P* < 0.05” and “*P* < 0.01,” respectively, and mean significant difference occurred after treatment.

Eighteen differentially expressed *GmWAK* genes were detected in soybeans under Cd stress. The changes in these genes in the treatment group were more than twice that of the control group (**Figure 13**). Six *GmWAK* genes were significantly upregulated under Cd stress in soybean roots. The expression of *GmWAK50/53* was increased by 3.7 and 1.7 times at 6 h after treatment, and *GmWAK1/48/52/71* increased by 3.7–8.1 times at 24 h. Eleven *GmWAK* genes were significantly downregulated in soybean roots. The expression of *GmWAK2/12/25/38/41/42/45/46/47* reduced by 67.8%–94.8% at 12 h after treatment. *GmWAK39/40* decreased by 83.1% and 89.5%, respectively, at 24 h. Nine *GmWAK* genes were significantly upregulated by Cd stress in soybean leaves. The expression of *GmWAK1/15/40/41/42/45/46* increased by 1.9–9.5 times that of the control at 6 h after treatment. *GmWAK2/12* increased to 5.4 and 3.4 times, respectively, after 24 h. Five *GmWAK* genes were significantly downregulated in soybean leaves. The expression of *GmWAK48* was reduced by 96% 3 h after treatment. *GmWAK25/47/50/71* were reduced by 65.6%–85.0% at 12 h. Among the 18 significantly differentially expressed genes, the relative expression of *GmWAK40/45/46* was at high levels in soybeans, which were significantly decreased by 67.8%–89.5% in the roots and increased by 2.7–5.0 times in the leaves.

## 4 Discussion

WAK is a family of receptor-like kinases crucial in signal transduction between the cell wall and the cytoplasm (Nakhamchik et al., 2004). WAK genes participate in several physiological processes in plant growth and development, such as cell expansion and elongation, pathogen resistance, and metal tolerance (Lally et al., 2001; Zuo et al., 2015; Xia et al., 2018). WAK genes are highly conserved. The typical domains of *WAK* genes include the transmembrane, EGF, GUB_WAK _bind, and Pkinase domains (He et al., 1999; Zhang et al., 2005; Zuo et al., 2019; Tripathi et al., 2021). At present, 27, 125, 6, 91, 29, 29, 29, 44, 68, and 27 *WAK/WAKL* genes have been identified in *Arabidopsis*, rice, wheat, barley, cotton, tomato, potato, apple, rose, and walnut, respectively (Vaid et al., 2012; De et al., 2014; Liu et al., 2006; Tripathi et al., 2021; Dou et al., 2021; Sun et al., 2020; Yu et al., 2022; Zuo et al., 2019; Liu et al., 2021; Li et al., 2022). However, the *WAK* gene family of soybeans has not yet been systematically identified and characterized. In this study, 74 *GmWAK* genes were identified in the soybean genome (**Figure 1**). There were more *WAK* genes in soybean than in *Arabidopsis* (27 genes) but much less than in rice (125 genes) (Vaid et al., 2012; De et al., 2014). This indicated a wide variation of the *WAK* gene family in plants. The expansion of the *WAK* gene family in plants has contributed to genome-wide duplication (Vaid et al., 2012; De et al., 2014). The increasing amount of *WAK* genes in soybeans was due to SD (27 genes), TD (15 genes), and PD (9 genes) events in genome-wide duplication (**Table 1**). The *WAK* gene family expanded through TD and large-scale duplication in *Arabidopsis*, whereas this gene family expanded through TD in rice (Shiu et al., 2004). This suggests differences among plants in the *WAK* gene family expansion. GD analysis showed that the TD events of *GmWAK* genes occurred approximately 23.84 Mya (5.88–52.4 Mya), the PD events of *GmWAK* genes occurred approximately 156.05 Mya (0.61–207.14 Mya), and the SD events were approximately 102.10 Mya (10.35–290.33 Mya) (**Table 1**). This result suggests that the expansion of the soybean *WAK* gene family is PD first, followed by SD, and finally TD.

Neofunctionalized or subfunctionalized genes occur in GD events (Lynch and Conery, 2000; Roulin et al., 2013). Neofunctionalization generated new functional genes, and subfunctionalization divided the functions of the ancestral gene (Conant et al., 2014; Gout and Lynch, 2015). The subfunctionalized genes coexisted on the genome and worked together in the dose-balance model to ensure the expression of genes at normal levels (Lan and Pritchard, 2016). The neofunctionalized gene pairs experienced positive selection (Ka/Ks > 1), while the subfunctionalized gene pairs experienced purifying selection (Ka/Ks < 1) (Lynch and Conery, 2000; Roulin et al., 2013). The Ka/Ks values of 34 duplication gene pairs were lower than 1 in the soybean *WAK* gene family, suggesting that these genes underwent purifying selection and might be subfunctionalized (**Table 1**).

The phylogenetic analysis showed that 74 *WAK* genes in soybeans were divided into four subgroups (**Figure 2**). Similar divisions were observed in other plants, such as *Arabidopsis*, tomato, barley, and cotton (Verica and He, 2002; Sun et al., 2020; Tripathi et al., 2021; Dou et al., 2021). Approximately 78.4% of *GmWAK* genes obtained three to four exons, and 16.2% of *GmWAK* genes obtained one to two exons (**Figure 3**; **Supplementary Table S2**). In other plants, 79.3%–89.7% of the *WAK* genes had three to four exons, and 3.5%–13.8% of genes had one to two exons (Sun et al., 2020; Dou et al., 2021; Yu et al., 2022). The structural similarity of *WAK* genes among different plants suggests that the *WAK* gene family was evolutionarily conserved. Additionally, *WAK* genes in the same subgroup had similar intron–exon distribution, conserved motifs, and domains, implying that they might share similar functions.

GUB_WAK_bind, EGF, transmembrane domain, and Pkinase domains are typical domains of the *WAK* gene family (Verica and He, 2002; Tang et al., 2022). The GUB_WAK_bind domain is rich in cysteine residues and constitutes the extracellular domain of Ser/Thr protein kinase, which can bind to cross-linked pectin, oligo-galacturonides, injury-induced pectin fragments, or pathogens, regulating cell expansion and activating stress responses (Ridley et al., 2001; Kohorn and Kohorn, 2012; Kohorn, 2015, 2016). The EGF domain is located in extracellular space. It is tightly connected to the cell wall and interacts with different proteins in the cell wall (Hu and Lou, 2010). The transmembrane domain transfers signals to downstream molecules through *in-vivo* phosphorylation (Verica and He, 2002). The Pkinase domain is located inside the cell and is the key domain in which the *WAK* gene functions (Verica and He, 2002. Fourteen *WAK* genes in the soybean *WAK* gene family possessed four typical domains, indicating that these genes might be able to perceive and recognize external environmental signals. Fifty-six *GmWAK* genes contained three typical domains (e.g., GUB_WAK_bind/EGF, transmembrane_domain, and Pkinase domain), and four *GmWAK* genes contained two typical domains (e.g., GUB_WAK_bind and Pkinase domain) (**Supplementary Table S2**). These genes included the Pkinase domain but lacked transmembrane or EGF domains, which might synergistically participate in extracellular signal transduction with other *WAK* genes. Nineteen hormone-responsive elements, 18 stress-response elements, 29 light-response elements, and 16 metabolic- and growth-related elements were identified in the promoter regions of soybean *WAK* genes (**Supplementary Tables S7**, **S8**). Hormone- and stress-related elements have been recognized in most *GmWAK* genes, such as MJA, JA, SA, ABA, ETH, GA, IAA, drought, heat, anaerobism, and trauma. MJA, JA, and SA are important signal molecules involved in plant pathogen defense. Such molecules improve plant resistance by promoting cell wall synthesis and maintaining cell wall integrity (Takahashi et al., 2004). ABA, ETH, GA, and IAA participate in plant growth, development, and abiotic stress response (Zhao et al., 2016; Luo, 2024; Colebrook et al., 2014; Xu and Chen, 2023). Previous studies showed that the *AtWAK* gene was highly expressed in vigorous growth and differentiation tissues when the cell size and morphology of the *Atwak* mutant of *Arabidopsis* changed (Wagner and Kohorn, 2001; Kohorn et al., 2006). *AtWAK1* showed a typical on-and-off pattern, with a first peak at 3 h and a complete disappearance after 9 h of Al exposure. Transgenic plants overexpressing *ATWAK1* enhance Al tolerance (Sivaguru et al., 2003). *ZmWAK* enhanced the resistance of maize to head smut disease (Zuo et al., 2015). Therefore, we speculate that the *WAK* gene family might be involved in the physiological process of plant growth and development and biotic and abiotic stresses by responding to multiple hormone signals.

The tissue expression pattern of a gene is closely related to gene function. Forty-seven *GmWAK* genes were expressed in different soybean tissues, such as the roots, stems, leaves, flowers, pods, and seeds (FPKM > 1) (**Figure 7**; **Supplementary Table S9**). Among them, the expression levels of six *GmWAK* genes were high: *GmWAK34/38/53* were highly expressed in the roots, *GmWAK40/45* were highly expressed in the stems or leaves, and *GmWAK15* was highly expressed in the roots, leaves, and pods. This result indicates that they might play an important role in the growth and development of soybean plants.

WAKs could perceive and transmit environmental signals to cells and play vital regulating roles in plant growth, development, and response to environmental stresses (Sun et al., 2020; Zhang et al., 2021). In this study, 12 *GmWAK* genes were expressed at high levels under abiotic conditions (**Figures 8** **–** **13**). Among them, *GmWAK40/45/46/47* were simultaneously observed in various abiotic stresses. *GmWAK40* was homologous with *AT4G03230* in *A. thaliana*. *AT4G03230* encoded a G-type lectin s-receptor-like serine/threonine-kinase, and its T-DNA insertion lines showed enhancing resistance to IAA, NPA, NaCl, and mannitol compared with the wild type (Ten et al., 2011). *GmWAK40* was highly expressed in soybean leaves. The expression levels of *GmWAK40* were significantly reduced by 76.1%, 94.9%, and 94.7% under cold, heat, and Al stresses, respectively. In contrast, they were significantly increased by 3.9 and 5.0 times after 3 h or 6 h, respectively, under salt or Cd stress. Many responsive elements, such as MJA, JA, ethylene, GA, cold, and heat, were identified in the promoter region of *GmWAK40*. MJA and JA are natural physiologically active substances and have been proven to regulate the adaptive responses of plants to various environmental stresses (Rahman et al., 2024). Multiple abiotic stresses were significantly induced by *GmWAK40*, suggesting that *GmWAK40* might be related to the response to different abiotic stresses. *GmWAK45/46* were a pair of duplicated genes that encoded the glycerol phosphodiesterases. Glycerol phosphodiesterase is a highly conserved enzyme in prokaryotes and eukaryotes, which participates in glycerol phospholipid metabolism by catalyzing glycerophosphodiester to generate glycerophosphate and alcohol (Patton-Vogt, 2007). Glycerol phospholipid metabolism participated in responding to various abiotic stresses in previous studies, such as the low nitrogen stress tolerance of sorghum, the cold stress of loquat, and the salt-alkali stress of *Suaeda salsa* (Wang et al., 2024; Xu et al., 2021; Sun, 2022). *GmWAK45/46* were highly expressed in soybean leaves. The expression of *GmWAK45/46* was significantly increased by 1.69–48.5 times under heat, salt, drought, and Cd stresses but was decreased by 72.5%–88.6% under cold and Al stresses. These results indicate that *GmWAK45/46* might participate in response to abiotic stresses with glycerol phospholipid metabolism. *GmWAK47* was homologous to *AT1G66980* in *Arabidopsis*. *AT1G66980* encoded an atypical receptor-like kinase, *SNC4*, which was a suppressor of *NPR1-1* and was related to the *Arabidopsis* defense response. Its mutant *snc4-1D* displays a constitutive activation of defense responses (Zhang et al., 2014). The expression of *GmWAK47* in soybeans showed significant changes under abiotic stresses. Decreases of 96.5% and 83.9% were observed under Al and Cd stresses, and an increase of 2.3–21.8 times was observed in the cold, heat, drought, and salt conditions. These results imply that *GmWAK47* is possibly involved in the response of plants to abiotic stresses.

## 5 Conclusion

We conducted genome-wide identification of the *WAK* gene family in soybeans in this study. Seventy-four *GmWAK* genes were detected in the soybean genome and unevenly distributed on 19 chromosomes. The 74 *GmWAK* genes could be divided into four subgroups based on phylogenetic analysis. The genes within the same subgroup shared similar gene structures, conserved motifs, and domains. SD was critical in expanding the soybean *WAK* gene family, and purification selection was conducted in evolution. Many hormone- and stress-response-related elements were predicted in promoter regions of the *GmWAK* genes. Eighteen *GmWAK* genes displayed significant changes under abiotic stresses. Among them, *GmWAK40/45/46/47* were simultaneously observed in various stresses, implying that they might be crucial for generating stress tolerance in soybeans. These genes could be potential candidates for investigating the molecular mechanisms of stress resistance in soybeans and as a genetic resource to cultivate new soybean varieties with enhanced stress resistance.

## Data Availability

The original contributions presented in the study are included in the article/**Supplementary Material**. Further inquiries can be directed to the corresponding authors.
